# A Giant Primary Angiosarcoma Invading the Right Heart in a Young Male: An Emergency Surgery

**DOI:** 10.7759/cureus.56309

**Published:** 2024-03-17

**Authors:** Hicham Elmalki, Mohammed Taha Berkane, Mehdi Moutaouekkil

**Affiliations:** 1 Cardiothoracic Surgery, Faculty of Medicine and Pharmacy, Laboratory of Anatomy, Microsurgery and Surgery Experimental and Medical Simulation (LAMCESM) Mohammed First University, Oujda, MAR; 2 Cardiothoracic Surgery, Mohammed VI University Hospital Center, Oujda, MAR

**Keywords:** infundibulotomy, open heart surgery, primary cardiac angiosarcoma, malignant tumors, right ventricular failure

## Abstract

Primary cardiac angiosarcoma is very rare. In this report, we describe an interesting case of a 25-year-old male with a giant primary angiosarcoma invading the right heart. He was urgently admitted to the hospital for respiratory distress. Once the diagnosis was suspected by chest x-ray, echocardiography, and CT scan, and given the patient's hemodynamic and respiratory instability, an emergency open-heart surgery was necessary to prevent complications. Through a right atriotomy and a pulmonary infundibulotomy, the tumor was resected. Invaded by the tumoral process, the tricuspid valve was replaced with a biological prosthesis. The postoperative course was marked by severe right ventricular dysfunction with multiorgan failure. Histopathologic examination of the surgical specimen confirmed a primary cardiac angiosarcoma.

## Introduction

Primary cardiac tumors are extremely uncommon; they can be classified into benign and malignant tumors. Benign lesions are the most frequent and account for 90% of all cardiac tumors [1]. Angiosarcoma is the most common primary malignant cardiac tumor [2]. The diagnostic methods have improved (echocardiography, CT angiogram, and cardiac MRI), but the management remains challenging, especially when the tumor extends to fundamental structures of the heart. In this study, we present a case of a primary cardiac angiosarcoma admitted to the cardiovascular surgery unit of the Mohamed VI University Hospital, Oujda, Morocco.

## Case presentation

**Figure 1:**
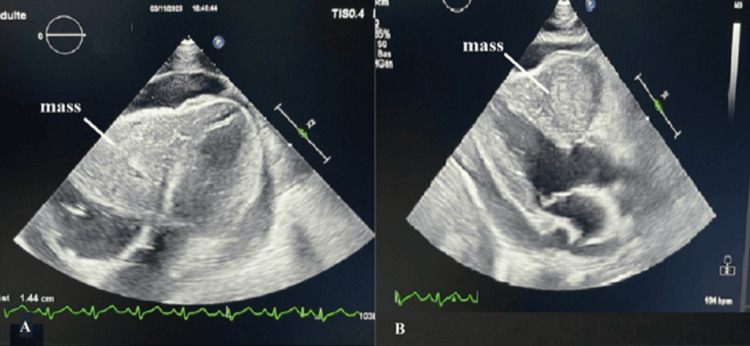
Echocardiographic appearance of the mass - (A) apical four chamber view and (B) parasternal long axis view.

A 25-year-old male, with no significant medical history, was admitted to the emergency department for dyspnea and chest pain. Clinical examination revealed a dyspneic patient, tachycardic, with a blood pressure of 90/50 mmHg, SpO_2_ 65% on ambient air and 83% on 15 L/min of oxygen, and marked cyanosis of the extremities with signs of heart failure, pulmonary auscultation revealed left basal crackles. Chest x-ray showed a cardiomegaly. Transthoracic echocardiography revealed a 46x68 mm mass filling the apex of the right ventricle with a base of implantation on the free wall, and extending towards the right outflow tract and towards the trunk of the pulmonary artery, we also note a protrusion of the mass in the right atrium (Figures 1A, 1B).

**Figure 2:**
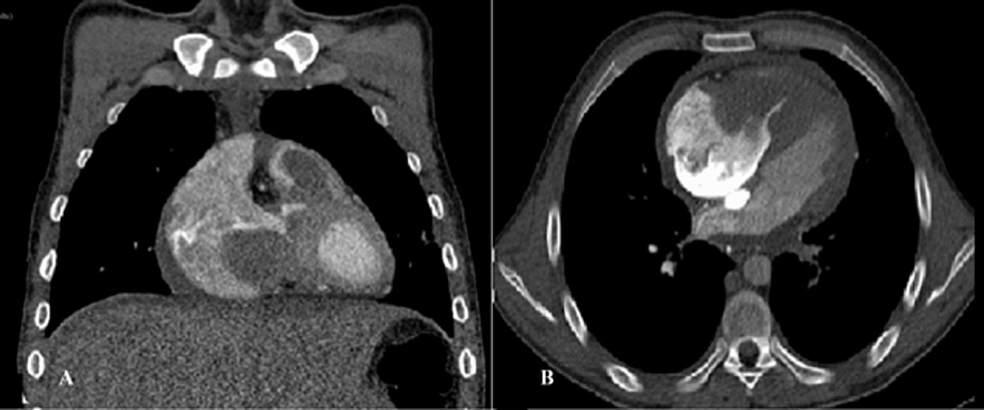
Cardiac multislice computed tomography showing a large mass occupying the right heart - (A) coronal view and (B) axial view.

**Figure 3:**
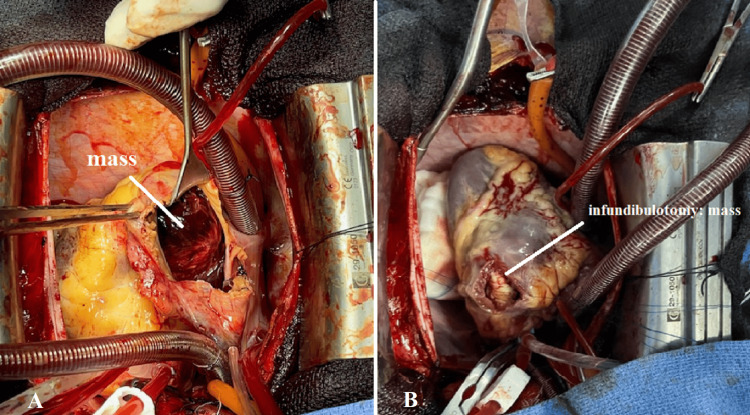
Surgical view after cardiac incisions showing the tumor - (A) right atriotomy and (B) infundibulotomy.

CT scan confirmed a large right intraventricular mass extending to the right atrium and to the origin of the pulmonary artery (Figures 2A, 2B). Given the patient's hemodynamic and respiratory instability, an emergency open heart surgery was performed through right atriotomy. An enormous tumor mass was discovered protruding into the right atrium through the tricuspid orifice, completely obstructing it (Figure 3A). The mass had a fleshy texture, somewhat friable. Subsequently, a pulmonary infundibulotomy revealed the same mass invading the walls of the right ventricle and the subvalvular tricuspid apparatus, protruding into the pulmonary infundibulum (Figure 3B).

**Figure 4:**
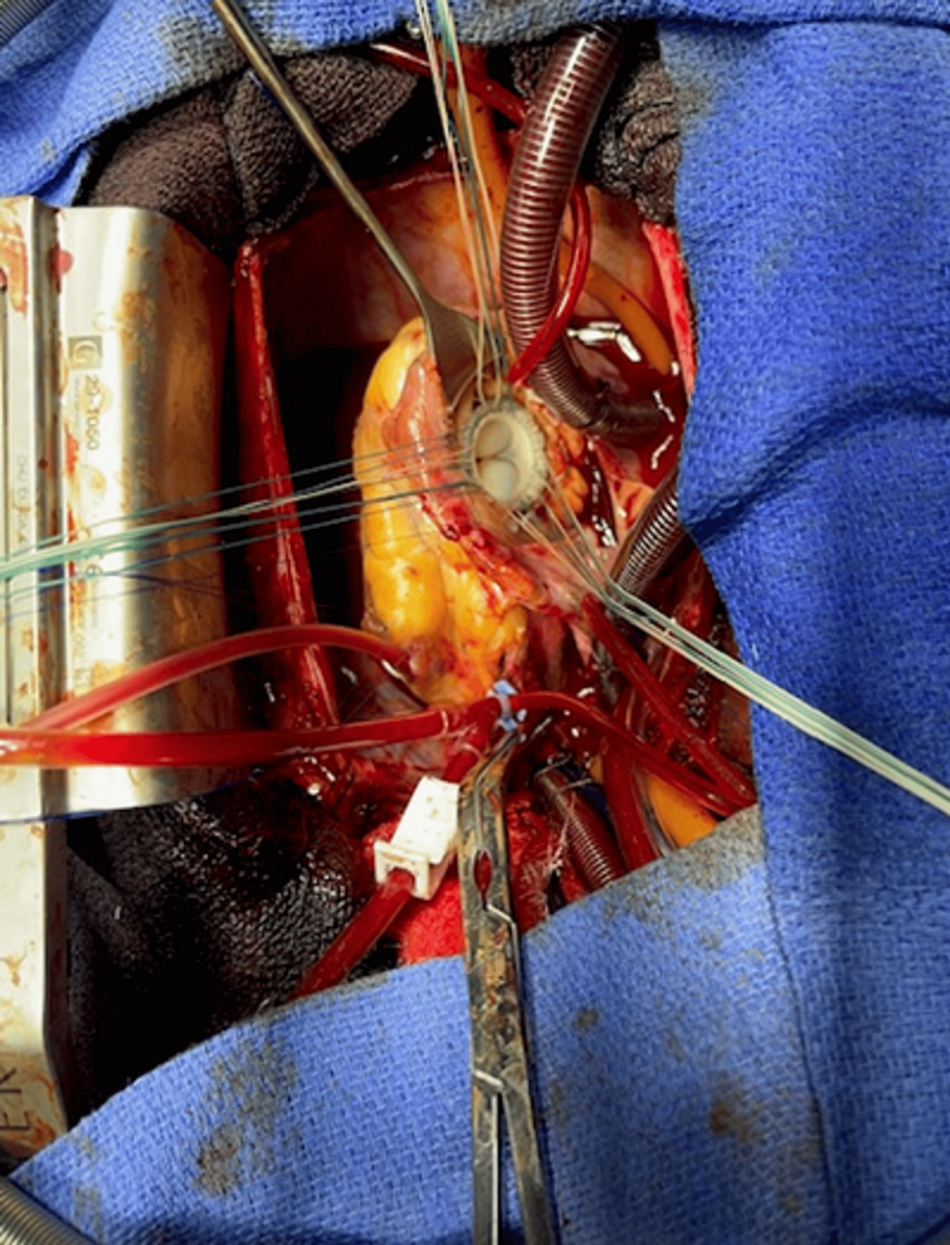
Surgical view showing the tricuspid valve replacement.

**Figure 5:**
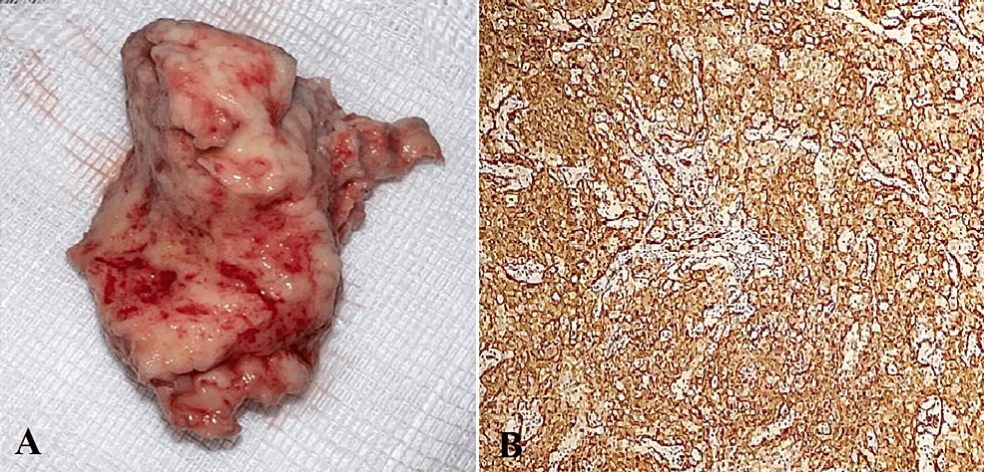
Macroscopic view of tumor (A) and CD31 diffuse positive marking of tumor cells confirming diagnosis of angiosarcoma (B).

The tumor was resected through step-by-step dissection, removing the tricuspid valve leaflets and a portion of the valvular apparatus. However, a portion of the tumor invading the tricuspid annulus, interventricular septum was left. The tricuspid valve was then replaced (Figure 4). The postoperative course was marked with severe right ventricular dysfunction with multiorgan failure. The patient passed away on the fourth day after surgery. Histopathologic examination of the surgical specimen later revealed a primary cardiac angiosarcoma (Figures 5A, 5B).

## Discussion

Angiosarcoma of the heart is an uncommon malignancy that develops from the endothelium within the heart's blood vessels. It constitutes approximately 25-30% of all primary malignant heart tumors and is acknowledged as the deadliest [3]. Although its primary site is often observed in the right cardiac chambers, especially the right atrium, cardiac angiosarcoma can also affect other heart components. Given its infiltrative characteristics and tendency for prompt metastasizing, diagnosing and treating this type of tumor presents substantial difficulties [3]. Due to its location in the heart, this type of tumor may present with diverse and non-specific signs frequently leading to a late diagnosis. Dyspnea is the most frequent sign, present in 59-88% of patients [4]. Other symptoms include chest pain, syncope, palpitations, and generalized fatigue [5]. Additional clinical presentations may involve valvular malfunction, pericardial effusion, or tamponade [3]. In certain instances, general symptoms due to secondary locations may be present. These include fatigue, weight loss, and a general sense of malaise. The lungs, liver, and lymphatic nodes are frequent sites of metastasis, substantially impacting the prognosis of the disease [6,7]. On echocardiography (both transthoracic and transesophageal), angiosarcomas typically present with an irregular mass, characterized by an immobile aspect, extending from the endocardium to the myocardium; pericardial effusion may be present as well [8]. On computed tomography, angiosarcomas are frequently depicted as irregular masses within the cardiac chambers, primarily affecting the free wall of the right atrium. Enhancement of these lesions after injection of contrast is heterogeneous, CT is also useful in the diagnosis of complications (i.e., pulmonary embolism) and systemic metastasis [9]. On cardiac MRI, we observe heterogeneous signal patterns on different sequences, reflecting the tumor and its local complications (necrosis and hemorrhage). Initial enhancement is noted at the arterial phase after which heterogenous enhancement is observed on late gadolinium enhancement (LGE) [9]. Confirmation of cardiac angiosarcoma typically requires a tissue biopsy. Cytology of pericardial fluid is considered unreliable, as tumor cells are rarely identified even in cases with pericardial infiltration [10]. Endomyocardial biopsy has shown limited diagnostic effectiveness [11]. Surgical biopsy after exploration or resection is recommended for accurate diagnosis [12]. Histopathological examination unveils specific histological characteristics of primary angiosarcoma, which include interconnected vascular channels and areas containing spindle cells [8]. Immunohistochemical analysis is crucial and involves positive staining of CD31 and CD34, which confirms that the tumor cells originate from the endothelium [13]. Differential diagnoses consist of cardiac metastasis, thrombus, endocarditis, foreign body, or other local tumors [14]. Surgery is the main therapeutic strategy for primary angiosarcoma, aiming for complete resection of the tumor. Opting for complete resection offers the greatest chance of prolonged survival [15]. The scope of the resection is determined by the tumor's size, location, and spread and may not be feasible in some cases deemed "inoperable" [3]. Other treatment options include chemotherapy, although many agents exist, a primary cardiac angiosarcoma chemotherapy regimen hasn't been established yet and the outcomes remain mediocre [3]. Radiation therapy may also be used in the management of primary angiosarcoma either as adjuvant or alone if surgical resection is deemed non-feasible [16]. The multidisciplinary approach, involving multiple specialists including cardiac surgeons, medical and radiation oncologists, radiologists, and pathologists, is necessary to manage these complex tumors.

## Conclusions

Primary angiosarcoma represents the most prevalent form of malignant primary cardiac tumors. Although uncommon, it is a very aggressive form of heart tumor arising in most cases from the right cardiac chambers typically the atrium. Its clinical presentation is very broad and non-specific. Diagnostic studies include echocardiography, CT, and cardiac MRI. Confirmation is acquired with a histopathological examination of surgical specimen. Surgical resection, if possible, is the most promising approach in terms of survival and potential curative outcomes. Chemotherapy and radiation therapy may also be used as adjuvant to surgery or as a palliative approach.
